# The olfactory pathway in the peracarid crustacean *Parhyale hawaiensis* (Malacostraca): new insights into the evolution of olfactory processing in Pancrustacea

**DOI:** 10.1098/rsob.240397

**Published:** 2025-05-07

**Authors:** Katja Kümmerlen, Rabea Schlüter, Steffen Harzsch

**Affiliations:** ^1^ Zoological Institute and Museum, University of Greifswald, Greifswald, Mecklenburg-Vorpommern, Germany; ^2^ Imaging Center of the Department of Biology, University of Greifswald, Greifswald, Mecklenburg-Vorpommern, Germany

**Keywords:** Peracarida, chemical senses, aesthetascs, olfactory receptor neurons, distributed olfactory coding, evolution

## Abstract

Our current understanding of the functional morphology of olfactory systems in arthropods largely relies on information obtained in hexapods. Existing analyses of the olfactory pathway in crustacean representatives have suggested that these animals share several corresponding anatomical elements with hexapod olfactory systems but that the latter likely feature a different olfactory wiring logic from receptor to olfactory glomerulus. This study sets out to further explore the diversity of arthropod olfactory systems by presenting a detailed morphological analysis of the peripheral and central olfactory pathways in an emerging model system, the peracarid crustacean *Parhyale hawaiensis* (Malacostraca). These animals feature all neuronal elements that characterize malacostracan crustacean’s olfactory systems, and the simplicity of this animal’s olfactory system provided the unique opportunity to quantify the numbers of olfactory sensilla and associated sensory neurons, olfactory interneurons and olfactory glomeruli. These data showed that the number of those neuronal elements is highly variable across individuals, contrasting with more stable numbers of neuronal elements in hexapod olfactory systems that typically are characterized by olfactory glomeruli with individual identities and constant numbers. We discuss the possible steps needed for an evolutionary transformation of a malacostracan crustacean type of olfactory system into a hexapod type.

**Figure:**
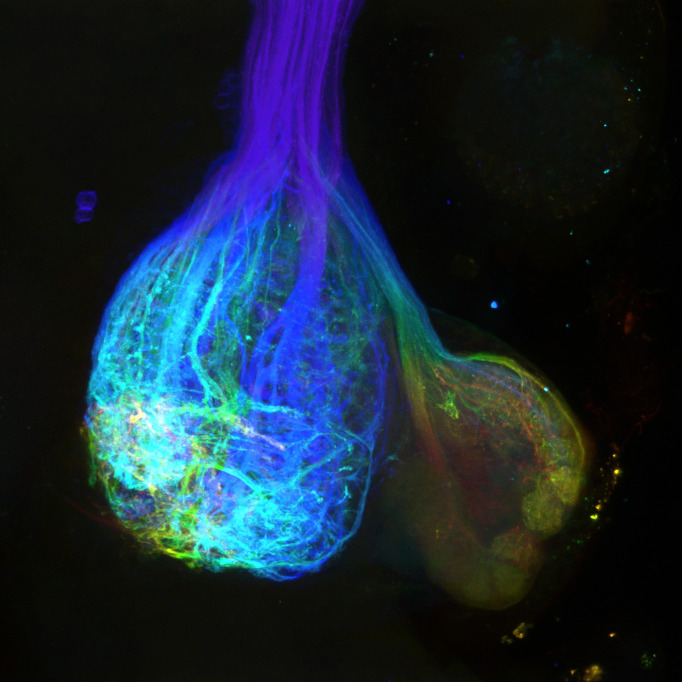

## 1. Introduction

It has long been known that, despite their wide phylogenetic separation, mammals such as mice and hexapods such as the vinegar fly *Drosophila melanogaster* have convergently evolved remarkably similar architectures of their olfactory systems and share corresponding principles in the logic of odour coding (reviews in e.g. [1–3]). Phylogenomic and ‘neurophylogenetic’ studies have firmly established a placement of Hexapoda as an in-group within the taxon traditionally known as ‘Crustacea’, together forming a clade termed ‘Tetraconata’ or ‘Pancrustacea’ [4–9]. Therefore, comparative analyses of olfactory systems across the Panarthropoda including, e.g., malacostracan crustaceans such as crayfish, crabs and lobsters offer the valuable opportunity to explore evolutionary transformations of olfactory processing mechanisms (reviewed in [10]). These animals live in a chemosensory landscape rich in chemicals from conspecifics, prey and predators, a fact that has led to the suggestion that chemoreception is a dominant sensory modality for most malacostracan crustaceans [11–13], which typically possess two parallel pathways for combined mechano- and chemosensory processing that were suggested to function as systems for ‘olfaction’ versus ‘distributed chemoreception’ (reviews in [11–14]). Malacostraca feature various setae or sensilla across their bodies, especially on the first and second antennae, mouthparts and legs (reviews in [15–18]). While bimodal setae are widely distributed, the specialized unimodal aesthetascs, dedicated only to olfaction, are confined to the first pair of antennae [10,12,18,19]. Aesthetascs contain branched dendrites of olfactory sensory neurons (OSNs) that vary in number across species and are located just beneath the cuticle [18,20–22]. Odorants are detected by ionotropic receptor molecules within the dendritic membranes of the OSNs [23,24], which project their axons into the primary olfactory centres, the olfactory lobes, where they contact the neurites of local olfactory interneurons and olfactory projection neurons in synapse-dense regions termed olfactory glomeruli (reviews in [10–12, 25]). In addition to the olfactory lobe, malacostracan crustaceans possess another primary sensory neuropil in their central olfactory pathway, the lateral antenna 1 neuropil (LAN), which receives input from bimodal (chemo- and mechanosensory) and mechanosensory setae on the first pair of antennae. While the olfactory lobe (OL) is thought to analyse odorant identity, the LAN is believed to extract directional information towards an odour source [12–14]. Large decapod (‘ten legged’) crustaceans such as crayfish, crabs and clawed lobsters have, for several decades, served as distinguished models to study the functional morphology of the crustacean olfactory system (reviews in e.g. [26–29]). However, information on the actual numbers of neuronal elements in the central olfactory pathway of Malacostraca remains scarce. The most comprehensive analysis is available for the spiny *Panulirus argus*, which possesses approximately 1 200 aesthetascs, each associated with around 350 olfactory sensory neurons, amounting to a total of 420 000 sensory neurons across the first antenna [30–33]. The number of olfactory glomeruli in this species is reported to range from 750 [30] to 1 100 [31], and the number of neurons associated with the OL is estimated to 100 000 local olfactory interneurons and to 200 000 olfactory projection neurons [31].

Sharing a common ancestor, hexapods and malacostracans exhibit several neuroanatomical similarities in their olfactory pathways [10], including spherical olfactory glomeruli and diverse types of local olfactory interneurons and olfactory projection neurons that target the mushroom bodies (reviewed in [25,34]). Nevertheless, notable differences exist between Malacostraca and Hexapoda (e.g. in the wiring logic from the receptor molecules in the OSNs to the olfactory glomeruli), and these differences were suggested to mirror different olfactory coding strategies [10,35]. In the hexapod model *D. melanogaster,* each OSN expresses a single type of olfactory receptor protein, and OSNs with the same receptor project to a dedicated olfactory glomerulus, giving each glomerulus a specific, individual identity. Odours are represented by a combinatorial code across the glomerular ensemble [36–42]. In contrast, evidence for a one-to-one connectivity from receptor to glomerulus so far is missing in malacostracan crustaceans (reviews in [10,23]). These animals possess only ionotropic receptors (IRs), with receptor diversity possibly arising from combinations of multiple IRs and co-receptors [23,43–45]. Furthermore, so far there is not any evidence for the presence of individually identifiable olfactory glomeruli in Malacostraca and OSNs appear to project to multiple and not to single glomeruli [10,23,46].

**Figure 1.**
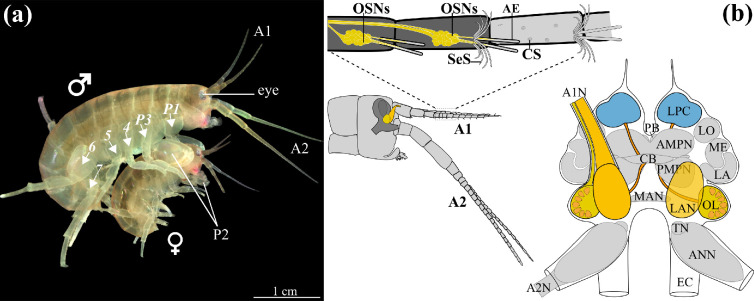
*Parhyale hawaiensis* and its olfactory pathway. (a) Picture of a male and a female *P. hawaiensis* in precopula (modified from [47], creative commons license). (b) Schematic drawing of the olfactory pathway in *P. hawaiensis*. Detail of the head and the first and second pair of antennae, with the brain contours superimposed. Detail of the first antenna, with the different types of setae on the first antenna and OSNs, associated with the aesthetascs, in yellow. Schematic representation of the brain of *P. hawaiensis* (modified from [47]; Creative Commons license), the neuropils important for olfactory processing are highlighted in colour. A1, antenna 1; A1N, antenna 1 nerve; A2, antenna 2; A2N, antenna 2 nerve; AE, aesthetascs; AMPN, anterior medial protocerebral neuropil; ANN, antenna 2 neuropil; CB, central body; CS, cuspidate setae; EC, esophageal connective; LA, lamina; LAN, lateral antenna 1 neuropil; LO, lobula; LPC, lateral protocerebrum; MAN, medial antennae 1 neuropil; ME, medulla; OL, olfactory lobe; OSN, olfactory sensory neurons; P1−7, pereopods 1−7; PB, protocerebral bridge; PMPN, posterior medial protocerebral neuropil; PNT, projection neuron tract; SeS, serrate setae; TN, tegmentary neuropil.

The present paper sets out to contribute new insights into diversity of olfactory systems in Malacostraca as an important clade within Arthropoda. In this study, we chose to analyse this question using the amphipod crustacean *Parhyale hawaiensis* (Dana, 1853, Peracarida, Amphipoda and Hyalidae; figure 1a) which belongs to the underexplored taxon of peracarid crustaceans, in contrast to the more extensively studied decapods. It is considered an emerging crustacean model species [48,49], a sequenced genome is available [50] and a number of genetic tools have been developed, including CRISPR (clustered regularly interspaced short palindromic repeats)-mediated gene editing [51]. Also, a detailed description of the brain was published [47], and an analysis of the neurochemical diversity in the central olfactory pathway is available [52]. Here, we provide a comprehensive neuroanatomical analysis of the olfactory pathway of these crustaceans from sensilla to olfactory glomeruli. Taking advantage of the (relatively) low numbers of neuronal elements in both peripheral and central olfactory pathway of this species (compare [52]), our study sets out to explore numerical aspects such as quantifying the olfactory sensilla, the olfactory sensory neurons and the olfactory glomeruli as well as the olfactory interneurons. What is more, stimulated by the knowledge on the hexapod olfactory system, we attempted to individually identify olfactory glomeruli. We compare our findings with Hexapoda, and based on the well-established phylogenetic relationships within Pancrustacea, discuss evolutionary transformations of selected aspects of olfactory system design in this clade.

## 2. Material and methods

*Parhyale hawaiensis* were reared in aquaria with aerated artificial seawater (32 PSU) at approximately 25°C, under a 12/12 h dark/light cycle. Animals were fed three times a week with JBL NovoCrabs (ad libitum), artificial seawater was changed every month. For all experiments, sexually mature males were used (compare [52]).

### 2.1. Immunohistochemistry

To label the olfactory glomeruli, we used immunohistochemistry against synapsins, a family of presynaptic proteins [53], following the methods laid out in [52]. Animals were cold-anesthetized and beheaded, the heads then fixed in 2% paraformaldehyde (PFA, Electron Microscopy Science, lot 511179307) in 0.1 M phosphate-buffered saline (PBS, pH = 7.2) and 5% glucose (Sigma-Aldrich, Burlington, USA) overnight at 4°C. Brains were carefully dissected, then washed with blocking buffer (PBT: PBS with 0.3% Triton X-100, TX, Sigma-Aldrich) and 1% bovine serum albumin (BSA, Sigma-Aldrich) at room temperature (RT) to decrease unspecific binding of antibodies in the tissue for 2 × 15 min, 2 × 30 min and 1 × 60 min. Whole-mounts were incubated in antisera against synapsins ‘SYNORF1’ (raised in mouse, developmental Studies Hybridoma Bank, IA, USA, #3 C11, RRID AB_528479) at a dilution of 1:10 in blocking buffer PBT for 3.5 days at RT with gentle agitation. Several washing steps (2 × 10 min and 2 × 20 min) were carried out with PBT at RT. Subsequently, brains were incubated for 2.5 days in the secondary antisera (goat anti-mouse Cy3, Jackson Immuno, Baltimore, USA, cat. no. 115-165-003, diluted 1:500 in blocking buffer) and a nuclear counterstain (either Hoechst 33342 Thermo Fisher scientific, Waltham, USA or Sytox Green, Invitrogen, Carlsbad, USA, both diluted 1:10 000 in blocking buffer). Brains were washed with PBT (2 × 10 min, 2 × 20 min and 1× overnight), infiltrated in a two-step series with PBS-glycerol (Carl Roth, Karlsruhe, Germany, 1:1 for 1 h and 3:1 glycerol–PBS for 20 min) and mounted in dabco-glycerol (Carl Roth).

To label olfactory sensory neurons, we used the iGluR1 antibody (Kerafast, Shirley, USA, EKY005). First antennae were removed, horizontally sectioned in three pieces and fixed for 5 min in 2% PFA in PBS with 5% glucose, then washed in PBS for 2 × 5 min and infiltrated with 9:1 glycerol:DMSO (Dimethyl sulfoxide, Carl Roth) for 30 min with gentle agitation at RT. Infiltrated first antennae were frozen twice in liquid nitrogen. After washing with PBT-BSA (2 × 15 min, 2 × 30 min), tissues were incubated in the secondary antibody solution (1:500 Cyanine3 goat anti-rabbit IgG, Invitrogen, cat. no. A10520) with a nuclear counterstain (1:10 000 Sytox Green, Carlsbad, Invitrogen). Tissues were then washed, infiltrated in PBS–glycerol and mounted on slides in dabco-glycerol.

### 2.2. Specificity of the antisera

#### 2.2.1. Synapsin

The monoclonal anti-*Drosophila* synapsin SYRNOF1 antibody (Developmental Hybridoma Bank (DSHB) Hybridoma Product 3C11, anti-SYRNOF1 as deposited to the DSHB by E. Buchner, University Hospital Würzburg, Germany, supernatant, AB_528479) was generated using *D. melanogaster* glutathione-S-transferase (GST)–synapsin fusion protein as antigen. The antibody is able to identify at least four different synapsin isoforms (70, 74, 80 and 143 kDa) in western blots of *D. melanogaster* head homogenates [53]. A western blot analysis performed on the brain tissue of *D. melanogaster* and the crustacean *Coenobita clypeatus* produced strong antibody-stained band around 80−90 kDa in both species [54]. In previous studies on *P. hawaiensis*, this antiserum labelled brain structures in a reproducible pattern consistent with the expectation that this antiserum labels synaptic neuropils [47,52,55]. However, because here no additional experiments were conducted to determine the specificity of this antiserum in *P. hawaiensis*; therefore we will refer to the labelling as ‘synapsin-like immunoreactivity’ (synapsin-like IR).

#### 2.2.2. Ionotropic glutamate receptor

The polyclonal anti-Lobster iGluR1 antibody (Kerafast, from the laboratory of Timothy S. McClintock, PhD, University of Kentucky) was raised in rabbit against two non-overlapping peptides TGEGFDIAPVANPW and EYPTNDVDKTNFN and recognizes the lobster (*Homarus americanus*) ionotropic glutamate receptor (iGluR1). The antiserum was affinity purified, and preadsorption of 20 µl antibody solution with 1 mg of each peptide followed by centrifugation completely abolished the staining [56]. Correlating antibody staining with data obtained from *in situ* hybridization suggested that in the first antennae of spiny lobsters this antiserum specifically labelled OSNs by recognizing a motif of the ionotropic co-receptor IR25a but not any other cell types [56,57]. Another study combining antibody labelling and *in situ* hybridization in spiny lobsters showed a broad expression of iGluR1 in OSNs and came to the conclusion that this antiserum labels most if not all OSNs in the first antennae [58]. This antibody was also successfully applied to label OSNs in the decapod crustacean *C. clypeatus* [59]. In all these studies, labelling was not only present in the OSN dendritic compartment but also in the soma. As a positive control of the antibody, we additionally stained first antennae of the crayfish *Procambarus virginalis,* a decapod like *H. americanus*. No additional experiments were conducted to determine the specificity of this antiserum in *P. hawaiensis* so that we will refer to the labelling as ‘iGluR1-like immunoreactivity’ (iGluR1-like IR).

### 2.3. Anterograde filling

Heads of *P. hawaiensis* were fixed for 1–5 days in 2% PFA in PBS with 5% glucose. DiI crystals (1,1′-dioctadecyl−3,3,3′,3′-tetramethylindocarbocyaninperchlorat, Sigma-Aldrich) were carefully placed in the first antenna, which was either proximally cut between the third and fourth segment or distally cut, and the last 3–5 segments were removed. Proximal preparations were incubated in PBS at 4°C for 5–14 days, distal preparations were incubated in PBS at 4°C for 21 days, so the dye had sufficient time to diffuse through the cell membrane into the brain.

### 2.4. Fluorescence microscopy

Immunohistochemical and filled preparations were scanned using a Leica TCS SP5 II confocal laser-scanning microscope (cLSM, Leica Microsystems, Wetzlar, Germany) equipped with an argon-, diode-pumped solid-state (DPSS)- and diode lasers and operated with the Leica Application Suite Advanced Fluorescence software (LAS AF v. 2.7). Overview scans of the whole brain were scanned with 20× at a resolution of 1 024 × 1 024 pixels, resulting in a pixel size of 0.76 µm and a *z*-depth of 0.63 µm. Details were scanned with a resolution of 2 024 × 2 024 pixels, resulting in a pixel size for the 40× of 0.19 µm and a *z*-depth of 0.30 µm and for the 63× magnification of 0.085 µm and a *z*-depth of 0.30 µm. The pinhole was adjusted according to the objectives and the staining intensity. The nuclear dye Hoechst and the autofluorescence of the cuticula were excited at a wavelength of 405 nm, and the emission spectra were measured at 410−480 nm. The nuclear dye Sytox green as well as the antibody AlexaTM488 were excited at 488 nm, and their emission spectra were measure at 500−550 nm. Cyanine3 and DiI were excited at 550 nm, the Cyanaine3 emission spectra were measured at 560−650 nm and the DiI emission spectra were measured at 650−800 nm. For all images, single optical sections as well as maximum *z*-projections were globally enhanced using contrast and brightness in ImageJ.

### 2.5. Transmission electron microscopy

Electron microscopy work was carried out in accordance to the protocols published in [60]. Briefly, specimens of *P. hawaiensis* were cold-anaesthetised, then dissected in ice-cold Karnovsky fixative for marine animals [61] (2.5% PFA, 2% glutaraldehyde Electron Microscopy Science, Hatfield, USA and 5% glucose in phosphate buffer). Then, the dissected brains and first antennae were fixed in Karnovsky fixative using a laboratory microwave (BioWave Pro, Ted Pella, Redding, CA, USA) in combination with a solid-state cooling unit (Steady Temp Pro Thero Cube, Ted Pella). The BioWave protocol consisted of three microwave pulses of 2 min each, operated at a power of 150 W with breaks of 2 min to allow for cooling of the sample. The maximum temperature of the sample chamber did not exceed 20°C during the whole microwaving process. Afterwards, specimens were washed three times with PBS. Specimens were post-fixed with 1% osmium tetroxide (Electron microscopy sciences, Hatfield, USA) with 1% potassium ferrocyanide (Carl Roth) phosphate buffer at room temperature for 2 h. Tissues were then washed three times for 10 min with distilled water, then dehydrated in a graded ethanol series consisting of 50, 60 and 70% ethanol, then *en bloc* contrasted with 1% uranyl acetate (SERVA) in 80% methanol, then the ethanol series was continued with twice 80, 90 and 96% ethanol and three times 100% ethanol, and then specimen were incubated twice for 7 min in propylene oxide. Samples were embedded using an ‘Embed812’ resin embedding kit (Science Services, Munich, Germany). Pre-embedding was done with previously frozen resin using the following steps: infiltration with 1:1 propylene oxide:resin for 1 h and 2:1 propylene oxide:resin for 12 h. Afterwards, samples were transferred to fresh resin and incubated in a vacuum heating cabinet (VacuTherm, Thermo Fisher Scientific, Waltham, MA, USA) at 40°C and 100 mbar for 3 × 3 min to remove air in the resin. The vacuum was released slowly between each step, and air bubbles were removed. Resin blocks with the samples were polymerized in a heating cabinet at 60°C for 48 h.

Blocks were trimmed with the histological sectioning knife Histo Jumbo Diamant (Diatome, Nidau, Switzerland). Sections were stained using toluidine blue (Carl Roth) to check for the right region in the sample, and trimming depth was adjusted accordingly. Ultra-thin sections were prepared using an Ultracut 45° (Diatome) in combination with an EM UC7 Ultramicrotome EM UC7 (Leica Microsystems, Wetzlar, Germany). Ultrathin sections were transferred onto a Formvar-coated 2 mm × 1 mm copper grid (Plano GmbH, Wetzlar, Germany) and examined either with the transmission electron microscope JEOL JEM 1011 (USA) with a digital camera (MegaViewIII, Olympus K.K., Tokio, Japan) using an aniTEM software package (iTEM Software, Whiteley, UK) or with a transmission electron microscope Zeiss LEO 906 (Carl Zeiss Microscopy Deutschland, Oberkochen, Germany) equipped with a wide-angle-2K Dual Speed CCD-camera (Tröndle, Moorenweis, Germany), operated by the ImageSP software.

### 2.6. Field emission scanning electron microscopy

Pairs of first antennae of 6 individual *P. hawaiensis* were removed and fixed in 4% PFA with 5% glucose in PBS for 10 min, then washed three times with filtered artificial sea water. Tissues were then fixed for 2 days in Karnovsky fixative for marine animals at 4°C, subsequently washed 3 times for 10 min with PBS (pH = 7.2, 0.1 M) and post-fixed with 1% osmium tetroxide phosphate buffer for 70 min at RT. Afterwards, tissues were dehydrated in a graded series of ethanol (concentrations 10%, 30%, 50%, 70%, 90% and 100%) with 10 min per step at RT. The specimens were kept in 100% ethanol until they were critical point dried using a critical point dryer (Leica EM CPD300, Leica Microsystems) and then mounted on aluminium stubs using a double-sided carbon-coated conductive tape, making sure to mount the appendages on different sides, to scan all types of setae. Samples were sputtered with gold/palladium (Quorum Q150T ES, Quorum, East Sussex, UK) and examined with a FE-SEM Supra 40VP (Carl Zeiss Microscopy Deutschland) using the Everhart–Thornley secondary electron (SE) detector and the in-lens detector at a 70:30 ratio at an acceleration voltage of 5 kV. All micrographs were edited using Inkscape v. 2.0, where the background was carefully clipped and blacked out.

### 2.7. Data analysis

For the analysis of the aesthetascs and other types of setae, six first antennae were imaged with the field emission scanning electron microscopy (FE-SEM). Aesthetascs are identified by their typical rod shape. For the analysis of the OSNs, 8 pieces of first antennae with in total 10 OSN clusters were reconstructed in AMIRA v. 6.0.1 (Thermo Fisher Scientific) to get an accurate count of the OSNs. For the analysis of the afferents of the OSNs, five ultra-thin cross-sections of the proximal end of the first antenna were counted using Cellpose 2.0 [62].

To map the input of the aesthetascs and other types of setae onto the brain, 12 proximal backfills and 3 distal DiI backfills were analysed using ImageJ v. 2.14.0 [63]. For 20 animals, aesthetascs on the first antennae were manually counted based on their autofluorescence as detected with the Nikon Eclipse 90i microscope and double-checked in cLSM overview scans of the first antennae at an excitation of 405 nm.

Olfactory glomeruli, discernible in synapsin-like immunoreactivity, were segmented manually using AMIRA in detailed scans of the olfactory lobes. In the samples immunolabelled for synapsins, every fifth plane was manually segmented, then interpolated. The selected volume was transferred into a data file called ‘material’, and the information was exported using the material statistics function. The data was analysed using R v. 4.0.2 [64]. For the analysis, 42 olfactory lobes were used. For seven olfactory lobes, the embedding media ‘Mowiol’ was used, which shrinks while hardening. To compensate for the shrinking artefact, the average volume of the glomeruli in glycerol embedded samples and in Mowiol embedded samples was calculated, from these averages, a shrinkage factor of 2.23 was calculated. The volume of the Mowiol glomeruli was then multiplied by the shrinkage factor.

For testing a possible correlation with body size, numbers total antennal elements, consisting of antennomeres and flagellomeres, numbers of aesthetascs and numbers of olfactory glomeruli, the total body length of 12 individuals was measured with a Nikon Eclipse 90i equipped with a camera (Nikon DS-Fi3) at 2× magnification. Measurements were taken with the polyline measurement tool of the imaging software NIS-Elements AR (Nikon, v. 5.02.03). Viewed from the side, the animals’ dorsal profile was traced from the base of the first antenna to the tip of the telson (compare [55]).

Antennomeres, flagellomeres and aesthetascs were counted using autofluorescence. Glomeruli were reconstructed in AMIRA v. 6.1 (Thermo Fisher Scientific) by labelling about five to eight optical sections per glomerulus. For each individual, the left and right first antenna and olfactory lobe were analysed, and finally the measurements were averaged.

To test for possible over-segmentation by the experimenter, we determined the median glomeruli volume of one olfactory lobe compared against the number of glomeruli within that lobe (electronic supplementary material, figure S1). For over segmentation, we would expect the average volume of the glomeruli to decrease with an increasing number of glomeruli. The linear relationship between the median volume of the glomeruli and the glomeruli number is *y* = 1719.07*x* + 42.038 (*R*² = 0.02362, *n* = 25). Since the spatial relationship is a positive one and the *R*² = 0.02362, we are confident in no over segmentation.

For an estimation of the numbers of local olfactory interneurons and olfactory projections neurons, the nuclei associated with 10 olfactory lobes were analysed in cLSM scans. The nuclear counter stain channel was split into a single channel in ImageJ. The image stack was cropped to the dimensions of the olfactory lobe, then all nuclei not associated with the olfactory lobe were masked using the freehand selection tool. The resulting stack was saved using the .tif format. Using Cellpose 2.0 [62,65], the ‘cyto’ model from the model zoo of the pre-trained neuronal network was trained to the *P. hawaiensis* nuclei dataset using 30 randomly selected single optical sections of multiple olfactory lobes.

### 2.8. Nomenclature

Phylogenomic studies [8,9,66] and research in neural cladistics/neurophylogeny (e.g. [6,7]), confirm that Hexapoda is nested within the clade traditionally known as ‘Crustacea’. The terms ‘Tetraconata’ [4,5] and ‘Pancrustacea’ were proposed to reflect this evolutionary relationship. Here, ‘malacostracan Crustacea’ refers to a monophyletic group excluding Allotriocaridea, which encompasses Hexapoda.

Hair-like cuticular structures (sensilla) on the first antennae are termed ‘setae’ [16], with ‘aesthetascs’ distinguished by their rod-like morphology, implying olfactory function. The functions of other setae types remain unknown and are classified by external morphology following Garm [16]: ‘simple setae’: smooth appearance, ‘serrate setae with denticles’: scaled appearance, ‘pappose setae’: feathered tip, and ‘cuspidate setae’: short, located in slight depressions.

We use ‘OSNs’ as defined by Krieger & Breer [67], distinguishing the whole cell from the olfactory receptor molecules in the membrane. The term ‘lateral protocerebrum’ [34] is used instead of ‘hemiellipsoid body/terminal medulla’. Brain images are oriented dorsally, with right hemisphere structures shown or mirrored for clarity. Morphological descriptions are based on body axis orientation, and we avoid traditional malacostracan cell cluster terminology [68] due to the soma layer concealing cell cluster boundaries in *P. hawaiensis* [47,52].

## 3. Results

We analysed the peripheral and central olfactory pathways in the amphipod *P. hawaiensis*, quantifying key elements. Our study includes descriptions of the setae on the first antennae, associated neurons, transmission electron microscopy imaging of first antennae nerve bundles, first antennae input mapping via mass backfills, three-dimensional reconstruction of olfactory glomeruli using synapsin staining and quantification of inter- and projection neurons around the olfactory lobe.

### 3.1. The first antennae bear a variety of setae

**Figure 2.**
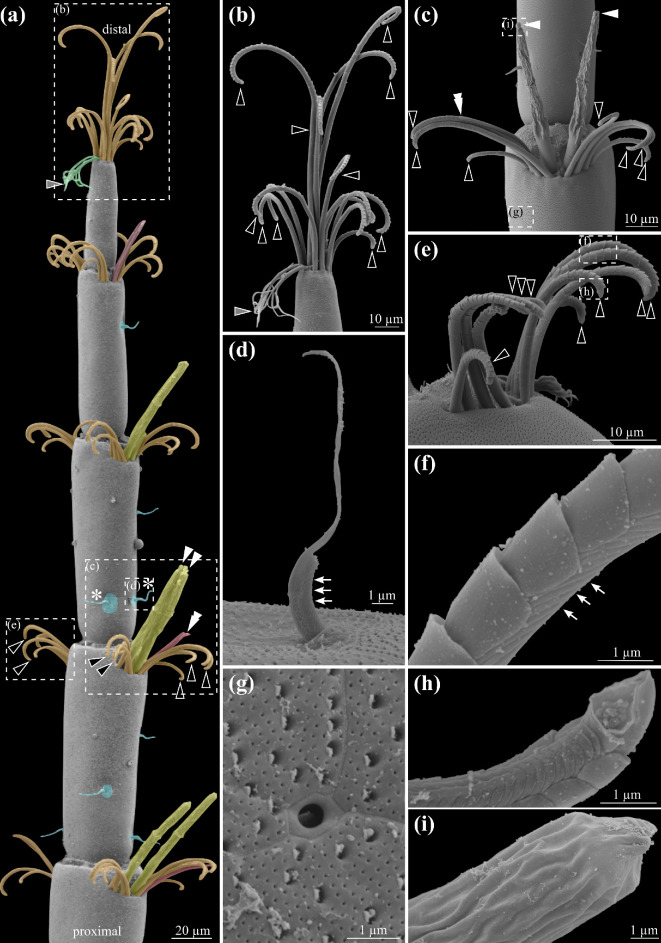
Scanning electron micrographs of first antenna. (a) Five most distal flagellomeres showing the pattern and diversity of the different types of setae of the first antenna. Yellow coloured structures and white arrowheads— aesthetascs; orange coloured structures and black arrowheads with white outline—serrate setae; red coloured structures and double arrowhead—simple setae; cyan coloured structures and asterisk—cuspidate setae; green coloured structures and grey arrowhead with white outline—pappose setae. The details are highlighted with dashed lines and a reference letter. (b) Detail of the tip of the antenna with serrate setae (black arrowheads with white outline), and pappose setae (grey arrowhead with white outline). (c) Detail of the aesthetasc cluster on the proximal end of most flagellomeres. Two aesthetascs (white arrowheads) surrounded by multiple serrate setae with denticles (black arrowhead with white outline) and one slender simple seta (white double arrowhead). (d) Detail of the cuspidate setae, arrows highlight the folds on one side of the sensilla. (e) Detail of the cluster of serrate setae with denticles (black arrowhead with white outline) on the opposite site to the aesthetascs and setae cluster. (f) Detail of the denticles and the folds (arrows) of the serrate setae, position indicated in (e). (g) Detail of the pore between the cuticle plates of the first antennae, position indicated in (c). (h) Detail of the terminal pore of the serrate setae, position indicated in (e). (i) Detail of the pore-less tip of the aesthetascs, position indicated in (c).

The first antennae of *P. hawaiensis* consisted of 17 ± 1.3 serially repeated elements, three proximal antennomeres and 14 flagellomeres, tapering in diameter distally (*N*_first antennae_ = 20, figure 2a). Using FE-SEM and confocal microscopy imaging of cuticular autofluorescence, five morphologically types of setae were identified: aesthetascs (figure 2a,c,i), serrate setae with denticles (figure 2a–c,e,f,h), simple setae (figure 2a,c), cuspidate setae (figure 2a,d) and pappose setae (figure 2a,b). In Malacostraca, the aesthetascs (figure 2c) were suggested to function in olfaction [17,18]. In *P. hawaiensis*, they were simple rod-shaped setae with a length of 60.6 ± 4.7 µm (*N*_aesthetascs_ = 20), a thin cuticle displaying weak autofluorescence (electronic supplementary material, figure S2) and a wrinkled appearance, lacking a terminal pore (figure 2i). Per first antennae, 21 ± 3 aesthetascs (*N*_first antennae_ = 20) were counted. Aesthetascs were always arranged in pairs on the distal end of every flagellomere and were absent on the three proximal antennomers, the first two and the last two flagellomeres. The aesthetascs were flanked by multiple serrate setae on both sides.

The most common type of seta on the first antennae was a serrate seta with denticles, present on every antennomere and flagellomere (figure 2a–c,e). The serrate setae were organized in groups of three to four setae on either side of the paired aesthetascs on the distal ends of the flagellomeres, continuing to the tip of the first antennae, and they were present at the opposite side to the aesthetascs, typically in clusters of six to eight. The serrate setae were slender, and their terminal half to third was covered in denticles with a scale-like appearance (figure 2b,c,e,f). The opposite side to the denticles had the appearance of regular folds (figure 2f, arrows, 2h). The serrate setae possessed one terminal pore (figure 2h). In the same cluster of setae surrounding the aesthetascs, one-to-two slender simple setae could be observed, characterized by a smooth appearance and no pores (figure 2a,c). We did not find any of the simple setae on the tip of the first antennae.

Cuspidate setae were observed in shallow depressions across all antennomeres and flagellomeres, located within a shallowly indented area of the first antennae (figure 2a,d). The cuspidate setae have stout appearance, with a wider base of approximately a quarter of their height. One side of the basal structure displayed folds (figure 2d, arrows), the opposite side was smooth and gave rise to a thin extension of the setae. On the tip of the first antennae, we found two pappose setae (figure 2a,b). They were characterized by multiple thin denticles that were arranged randomly and unique to the first antennae tip. We did not find similar types of setae on other areas of the first antennae.

### 3.2. The number of olfactory sensory neurons per cluster is variable

**Figure 3.**
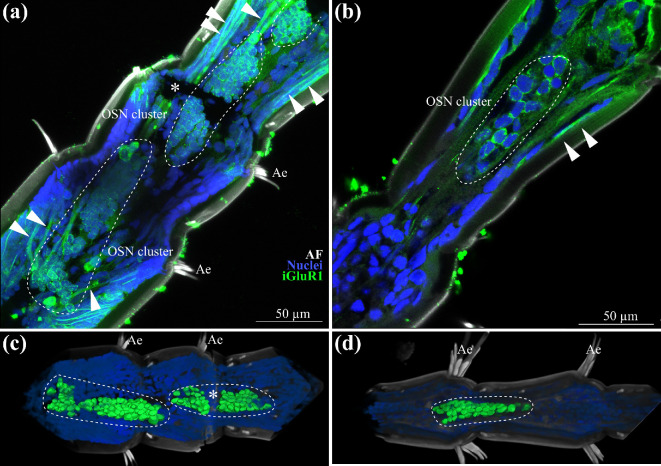
Olfactory sensory neurons. (a) Maximum projection of multiple optical sections (23.4 µm thickness) of iGluR1 labelling (green), counterstaining of the nuclei (blue) and cuticular autofluorescence (grey). Two OSN clusters are highlighted through the dashed line. White arrowheads highlight multiple dendrites. Asterisk indicates tear in antennae. (b) Single optical section (0.3 µm thickness), iGluR1 staining (green), showing one OSN cluster highlighted with dashed line, counterstaining of nuclei (blue) and cuticular autofluorescence (grey), dendrites highlighted with white arrowheads. (c) AMIRA reconstruction of two clusters of olfactory sensory neurons (green), highlighted with dashed line, the surface of every OSN is shown, overlaid with nuclei (blue) and cuticular autofluorescence. Asterisk indicates tear in antennae (d) AMIRA reconstruction of one cluster of olfactory sensory neurons, highlighted with dashed line (green), the surface of every OSN is shown, overlaid with nuclei (blue) and cuticular autofluorescence (grey). AF, cuticular autofluorescence; Ae, aesthetascs; iGluR1, ionotropic olfactory co-receptor in the membrane of the olfactory sensory neurons; OSN, olfactory sensory neurons.

To determine the number and location of neurons linked to aesthetascs, we stained for the iGluR motif of the ionotropic co-receptor IR25a using an antiserum that labels the majority of OSNs in lobsters [56–59]. In *P. hawaiensis*, using double-labelling with a DNA marker, we successfully identified multiple cell clusters and their dendrites consistent with an interpretation that this antiserum also labels at least a subset of the OSN in this species (figure 3; electronic supplementary material, figure S3). These clusters were typically located between flagellomeres, about half the length of a flagellomere away from their corresponding aesthetasc pair, probably receiving information from two pairs of aesthetascs (figure 3). Dendrites extended on both sides of the flagellomeres, despite aesthetascs being present on only one side. To test if the OSN number is related to the position along the proximal–distal axis of the antennule, the diameter of the widest part of the antennomere as a proxy for spatial position was related with the OSN number per cluster. We found no significant correlation between antennomere diameter and OSN count per cluster (*y* = 1.1121*x* + 17.118, *R*² = 0.3357). The clusters averaged at 112.2 ± 29.2 OSNs per cluster (*N*_OSNs_ = 10), ranging from 84 to 158 OSNs per cluster. We found one cluster per pair of aesthetascs, indicating an average of 56 OSNs per aesthetasc. Given 21 ± 3 aesthetascs per first antenna (*N*_first antennae_ = 20), we estimate about 1 178 ± 307 OSN axons per first antennae projecting to the brain.

### 3.3. Quantification of the afferents from the first antennae

**Figure 4.**
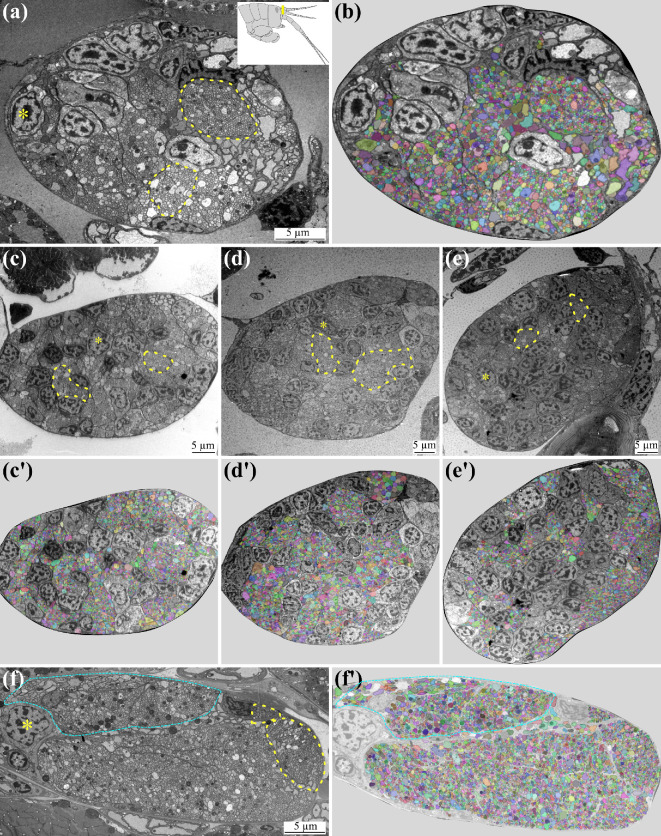
Ultrastructure of antenna one nerve for counts of axons. (a) Ultra-thin section of a cross section through the proximal end of the first antenna, showing the first antenna nerve. Schematic drawing of the head of *Parhyale hawaiensis*, cross-section indicated in yellow. (b) Counted axon profiles highlighted in multiple colours. (c –f) cross section of additional first antenna nerves of four more individuals. (c′–f′) visualization of counted axon profiles of the first antenna nerves. (f,f’) Prominent subsection of nerve highlighted in cyan. Asterisks—nuclei, yellow dashed line—dendrite bundles wrapped in myelin sheet.

**Figure 5.**
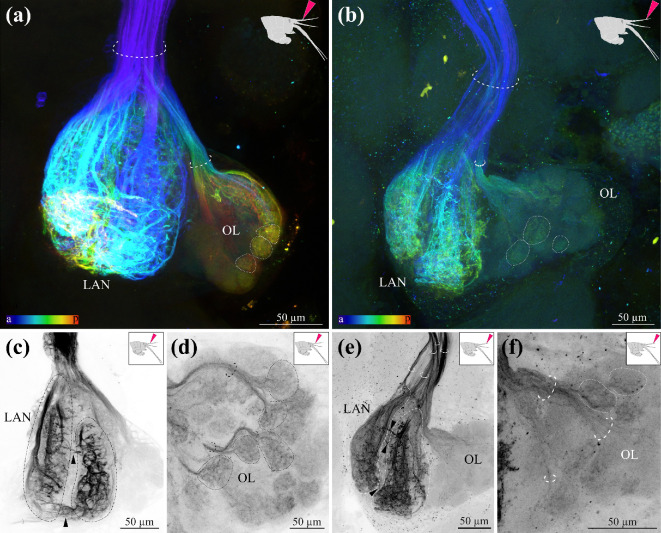
Mass anterograde filling of first antennae with neuronal tracer. (a) Depth-coded maximum projection (thickness 74.7 µm) of a proximal backfill from the third segment of the first antennae. Fibre bundles are indicated with dashed half circle, examples of olfactory glomeruli are indicated with dotted lines. Schematic insert of the *Parhyale hawaiensis* head shows spatial location of backfill. (b) Depth-coded maximum projection (thickness 35.7 µm) of a distal backfill from the third distal-most segment of the first antennae. Fibre bundles are indicated with dashed half circle, olfactory glomeruli are indicated with dotted lines. Schematic insert of the *P. hawaiensis* head shows spatial location of backfill. (c) Maximum projection of the LAN of a proximal backfill (thickness 3.6 µm). Fibre bundles are indicated with dashed half-circles, two-lobes structure of the LAN is indicated with dash-dotted line. Connections between the two lobes are indicated with arrowheads. (d) Maximum projection of the OL of a proximal backfill (thickness 7.5 µm). Fibre bundles are indicated with dashed half-circles, glomeruli are indicated with dotted lines. Gap in the dotted line shows the entry of the fibres into the cap region of the glomeruli. (e) Maximum projection of the LAN of a distal backfill (thickness 17.7 µm). Fibre bundles are indicated with dashed half-circles, two-lobes structure of the LAN is indicated with dash-dotted line. Connections between the two lobes are indicated with arrowheads. (f) Maximum projection of the OL of a distal backfill (thickness 18.3 µm). Fibre bundles are indicated with dashed half-circles, glomeruli are indicated with dotted lines. Gap in the dotted line shows the entry of the fibres into the cap region of the glomeruli. A, anterior; LAN, lateral antenna 1 neuropil; OL, olfactory lobe; p, posterior.

**Figure 6.**
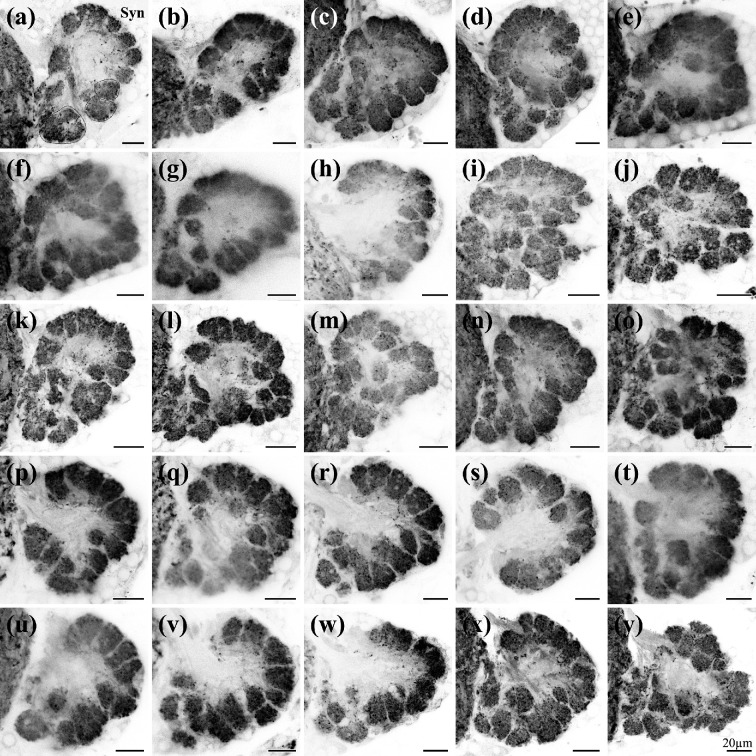
Synapsin staining of olfactory lobes in 25 individuals. Single optical sections (thickness 0.3 µm) of synapsin staining of olfactory lobes. Examples of olfactory glomeruli are indicated with dashed lines in (a). Optical sections are from a middle plane of the olfactory lobes. All images are oriented with dorsal towards the top, anterior towards the viewer and left olfactory lobes mirrored to show the right side. Syn, anti-synapsin staining.

Furthermore, we were interested in the numbers of the axonal projections of the OSNs as well as the other sensory neurons on the first antennae into the brain. Therefore, we cut the first antennae proximally (figure 4a′) and analysed ultrathin sections of the sensory neuron afferents of five adult male individuals. The nerve of the first antennae was wrapped in connective tissue, possibly a myelin sheet (figure 4a,c–f). Within the nerve, multiple axon groups wrapped in myelin were apparent (figure 4a,c–f, yellow dots). We also found multiple nuclei in all analysed nerves (figure 4a,c–f, asterisk). We counted an average of 4 621 ± 885 axons per nerve (*N*_nervs_ = 5, figure 4b,c′–f′), the number of axons ranging from 3 589 (figure 4c’) to 6 055 (figure 4b). Within one of the nerves, a prominent compartment was visible, possibly being the axons associated with the olfactory sensory neurons (figure 4b′). The number of axons in this compartment is 1 037 (figure 4b′).

### 3.4. Proximal and distal anterograde fillings showed similar results

Mass anterograde fillings from either the distal segments or the proximal segments of the first antennae were conducted to visualize the input from the first antennae into the brain (figure 5). The antenna 1 nerve was subdivided into multiple fibres, most likely axons, regardless of the position of the fill (figure 5a,b,e, dashed half-circles). The LAN was innervated by the vast majority of the axons in the nerve. These seemed to have a larger diameter than the axons innervating the OL, as the staining in the LAN was very prominent, structured and highly intense (figure 5a*–*d). Even though the staining was less prominent in the distal compared with the proximal fills, the same structures were labelled in both types of fill in the LAN (figure 5a*–*d). The LAN was subdivided into two elongated lobes (figure 5c,e, dash-dotted line) and had axon bundles innervating both lobes from dorsal to ventral (figure 5c,e). Within each lobe, horizontal profiles were apparent, and also between both lobes (figure 5c,e, arrowheads). In a number of proximal fills, we could observe a thin axon bundle exiting the LAN and projecting towards the midline of the brain, into the direction of the medial antenna 1 neuropil (figure 5c).

The OL was innervated by a separate set of fibres, probably axons from the olfactory sensory neurons, which split from the antenna 1 nerve in close proximity to the OL—this pattern was apparent in both distal and proximal anterograde fillings (figure 5a,b). Multiple olfactory glomeruli were clearly stained and delineated from each other in both the distal and proximal anterograde fillings (figure 5a,b,d,f, dotted lines). In both types of fills, the axon bundle split into smaller bundles (figure 5d,f, dotted half-circles), the axons in which targeted the glomeruli from the top (figure 5d,f, gaps in the dotted lines). Whereas in the proximal fills a large majority of glomeruli were stained, the distal fill visualized fewer innervated glomeruli (figure 5a,b,d,f). However, in the distal fills, more than half of the glomeruli were labelled, and no regionalization or clustering of stained olfactory glomeruli within the olfactory lobe could be observed (figure 5b,f), indicating that the position of the glomeruli does not correlate with the proximal versus distal position of the olfactory sensory neurons.

### 3.5. Olfactory glomeruli are heterogeneous in morphology and number

**Figure 7.**
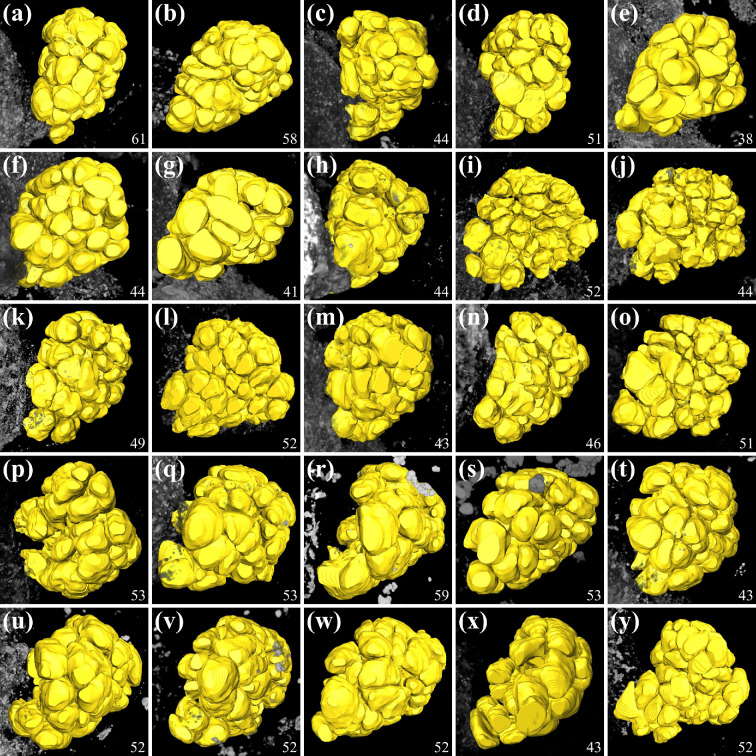
Three-dimensional reconstruction of olfactory glomeruli in 25 individual olfactory lobes. Surface view of AMIRA reconstruction of the same olfactory lobes shown in image figure 6 (letters correspond). The number of olfactory glomeruli is indicated in the bottom right corner. All images are oriented with dorsal towards the top, anterior towards the viewer and left olfactory lobes mirrored to show the right side.

**Figure 8.**
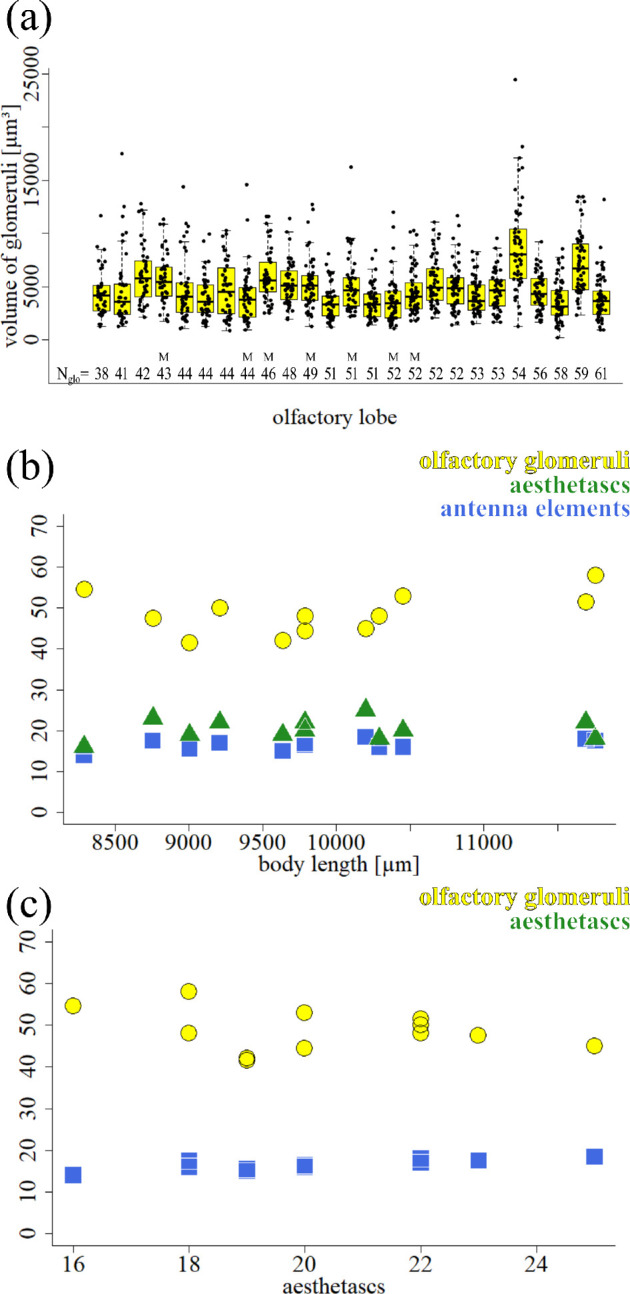
Volume of olfactory glomeruli, relationship to body length-aesthetascs. (a) Boxplot showing the average volume (in µm³) for the glomeruli within 25 individual olfactory lobes. The volume of every glomerulus is overlaid as black dots. The number of glomeruli within every olfactory lobe is stated in *N*_glo_. All olfactory lobes embedded in glycerol except for seven samples embedded in Mowiol, indicated with ‘M’ (b) Relationship between the body length (µm) of 12 adult males and the number of olfactory glomeruli (yellow, dots), the aesthetascs (green, triangles) and the antennomeres (blue, squares). (c) Relationships between aesthetascs and glomeruli (yellow, dots), and between aesthetascs and antennomeres (blue, squares).

Next, we wanted to gain more insights into the morphology and numbers of olfactory glomeruli in the olfactory lobe, specifically asking the question if fixed glomerular numbers are present in this species and if we can find glomeruli with individual identities based on spatial position. The basis for this analysis was antibody labelling of synaptic proteins, which resulted in a clear visualization of all neuropils (figure 6a–y; electronic supplementary material, figure S4a–q). The olfactory lobes had an ovoid shape and were subdivided in roughly spherical areas of neuropil, the olfactory glomeruli. However, even though the general shape of the olfactory lobe is very similar between individuals and also between the left and the right side of the same individual, the number, shape and spatial orientation of the individual olfactory glomeruli did not display any recognizable stereotypic pattern between individuals or between the left and right side of the same individual (figures 6a–y and 7a–y; electronic supplementary material, figures S4a–q and S5a–q). The total number of olfactory glomeruli was 48.5 ± 5.8 (*n* = 42). The lowest number of olfactory glomeruli in one olfactory lobe was 38, the highest number 61 (figure 8a). Furthermore, within the 42 analysed olfactory lobes of adult males, we encountered a high variability in the spatial arrangement of the glomeruli that made it impossible to attribute an individual identity to any of the glomeruli based on a stereotypic position in the lobe. The neuropil volume of the individual glomeruli was also highly variable: 4 905 ± 2 695 µm³ (*N*_glomeruli_ = 1 235).

We then tried to find possible correlations for the observed variability of glomerular numbers with other factors. Using the animal’s total body length as a proxy for age, we related the body length to the mean number of olfactory glomeruli of the left and right OL, the mean number of aesthetascs on both the left and right first antennae and the mean number of antennomeres of the left and right first antennae. We did not find a statistically significant linear relationship between the body length and the number of olfactory glomeruli (*y* = 0.002*x* + 30.220, *R*² = 0.1545, *n* = 12; figure 8b). We also did not find any statistically significant linear relationship between the number of aesthetascs and the body length (*y* = 0.0001*x* + 17.8662, *R*² = 0.01103, *n* = 12; figure 8b) and no statistically significant linear relationship between the body length and the antennomeres and flagellomeres (*y* = 0.001*x* + 9.842, *R*² = 0.3045, *n* = 12). We also tested if the number of glomeruli was related to the number of aesthetascs. We did not find a linear relationship between the aesthetasc numbers and that of the glomeruli (*y* = 60.5660*x* − 0.5873, *R*² = 0.08749, *n* = 12; figure 8c). We also did not find any linear relationship between the aesthetasc numbers and the total number of elements of the first antennae (antennomeres and flagellomeres in total) (*y* = 8.1014*x* + 0.4151, *R*² = 0.6501, *n* = 12; figure 8c).

### 3.6. Numbers of olfactory interneurons and projection neurons displayed inter-individual variation

**Figure 9.**
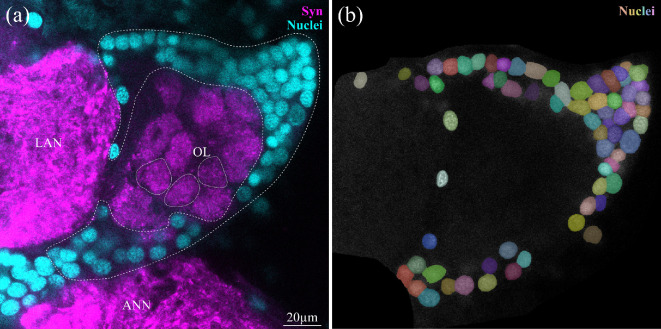
Olfactory interneurons and projection neurons. (a) Single optical section (thickness 0.3 µm) of an anti-synapsin labelling (Syn, magenta) and a nuclear stain (cyan). Olfactory glomeruli are indicated with dotted lines, inter- and projection neurons are indicated with dashed lines. (b) Single-channel image of nuclear stain of the inter- and projection neurons, with every nucleus marked by cellpose. ANN, antenna 2 neuropil; LAN, lateral antenna 1 neuropil, OL, olfactory lobe.

To complete our analysis, we quantified the number of the local interneurons and olfactory projection neurons associated with the OL. As pointed out in [47], in *P. hawaiensis* the local interneurons and projection neurons cannot be divided into two separate clusters (as is the case in decapod crustaceans [68]), therefore they were quantified together (figure 9). We counted 626 ± 97 (*N*_OLs_ = 10) inter- and projection neurons surrounding the olfactory lobe, ranging from 424 to 750 neurons. Even within the same brain, the neuronal numbers differed between left and right hemisphere (*N*_brains_ = 3, differences of 4, 39 and 170 neurons).

## 4. Discussion

### 4.1. *Parhyale hawaiensis* has an inventory of sensilla typical for malacostracan crustaceans

*Parhyale hawaiensis*, like other malacostracans, possesses two pairs of antennae. This study focused on the first pair (antennule), known to be equipped with bimodal chemo- and mechanosensory sensilla and the aesthetascs as typical olfactory organs [15–18]. We identified rod-shaped aesthetascs with a thin, wrinkled cuticle, similar to those in other malacostracans [18,69]. Although no moulting pore [70] was found, the rough texture of these sensilla’s tip may relate to its function because the weakly autofluorescent and wrinkly appearance of the aesthetasc cuticle likely mirrors a selective permeability to small molecules [71]. *Parhyale hawaiensis* displayed an average of 21 ± 3 aesthetascs per first antenna, for the isopod *Saduria entomon*, a number of about 60 aesthetascs were reported [72], and the mysid *Antromysis juberthiei* possesses between 14 and 21 [73,74]. These relatively low numbers in representatives of the Peracarida contrast with decapod crustaceans some of which possess up to 1 500 aesthetascs per first antenna (summarized in [10,75,76]). Unlike other Malacostraca [77–80], we did not find any correlation between body size and aesthetasc numbers in our study.

In addition to aesthetascs, we identified other types of setae, including serrate setae, simple setae, cuspidate setae and pappose setae that are also characteristic for other malacostracan crustaceans (reviewed in [16,17]). While their exact functions remain speculative without physiological or behavioural data, functional inferences can be drawn from morphological comparisons. Bimodal chemo- and mechanosensory setae, which are characterized by distal pores and which house dendrites for both mechano- and chemosensory neurons, are the most commonly observed setae on antennae [81,82]. Serrate setae, the most common type on the antennules of *P. hawaiensis*, likely function as bimodal sensilla as suggested by the distal pore (figure 2h) and morphologically similar serrate setae with mechanosensory function on the legs of *P. hawaiensis* [83]. Within the cluster of aesthetascs and serrate setae, we also identified simple setae, which lack a distal pore and are therefore unlikely to be bimodal sensilla but rather have a mechanosensory function. Additionally, we frequently observed cuspidate setae, which lack a distal pore and bear morphological similarity to mechanosensory microsetae found on the legs of *P. hawaiensis* [83]. Last, we observed prominent pores distributed across the surface of the first antennae (figure 2g), which perhaps have a secretory function akin to the tegumental glands observed in spiny lobsters [84]. Overall, the inventory of sensilla on *P. hawaiensis* first antennae mirrors that commonly identified in other malacostracan crustaceans suggesting that this species represents a typical malacostracan olfactory system.

### 4.2. The number of olfactory sensory neurons associated with an aesthetasc is variable

Associated with the aesthetascs, we identified an average of 112 ± 29 OSNs per cell cluster/sensillum pair, amounting to a total number of approximately 1 200 OSNs per first antenna so that we estimate that each aesthetasc is innervated by roughly 55 OSNs. This situation is comparable to that observed in other peracarid species, such as the mysids *Neomysis integer* and *Praunus flexosus*, where 25–40 OSNs innervate each aesthetasc [18,74]. Furthermore, in *P. hawaiensis*, we observed that OSNs are situated at some distance from the base of the aesthetascs, a feature also noted in the prawn *Macrobrachium rosenbergii* [18]. In larger decapods, such as the spiny lobster *P. argus*, up to 320 OSN dendrites per aesthetasc have been reported [85]. Our current thinking is that a crustacean’s entire chemoreceptive range is represented by the OSNs housed in each individual aesthetasc sensillum so that higher OSN numbers per aesthetasc may indicate that these animals can discriminate a wider spectrum of odorants [75]. Therefore, a multiplication of sensilla raises primarily the sensitivity of the system but does not promote the diversity of detectable odorants (reviewed in [10]). Without any information on the repertoire of olfactory receptor molecules in *P. hawaiensis,* this hypothesis requires careful evaluation. Transcriptomic studies in *P. argus* showed a universal expression of co-receptors IR25a and IR93a across all OSNs [44]. These co-receptors, with ‘tuning ionotropic receptors’, form complexes for detecting specific odorants [23]. Individual OSNs in *P. argus* may express multiple tuning IRs, suggesting potential for broad odorant detection or multimeric receptor formation [23,44].

So far, information on the ultrastructural organization of the antenna 1 nerve was only available for the spiny lobster *P. argus* [19,86]. In this species, the nerve consists of a mix of sensory and motor components. Shortly before it enters the brain, this nerve is partitioned into four major divisions with the smallest and most ventral of these divisions consisting of motoneuron axons, whereas the axons in the remaining three (medial, lateral and dorsal) divisions are sensory. In this species, large-diameter axons (10–50 µm) and smaller-diameter axons (1–5 µm) projected into the lateral antenna 1 neuropil, whereas thin to medium-sized, electron-dense axons projected into the OL [19,86]. In *P. hawaiensis*, we did not find these four major divisions but numerous smaller axon bundles, each wrapped by an electron-dense sheath. We counted 4 621 ± 885 axons in the nerve cross sections. Because we counted 1 178 ± 307 OSN associated with the aesthetascs, we can estimate that approximately 3 400 axons in the nerve belong to the sensory neurons associated with non-aesthetasc sensilla.

### 4.3. Anterograde filling with neuronal tracers—somatotopic representation in the lateral antenna 1 neuropil versus odotopic representation in the olfactory lobe?

Proximal and distal backfills of the first antennae in *P. hawaiensis* successfully stained the first-order chemosensory neuropils in its brain, LAN and OL. The axon bundle innervation the OL comprised characteristic fine neurites, as previously reported from the decapod *P. argus* [19,86]. In both type of preparations, olfactory glomeruli were innervated from the outside, the ‘cap’ region, by dense fibre networks, which again is consistent with reports on decapods [19,46,87,88]. Leucine uptake studies on crayfish indicated that marking few aesthetascs suffice to label all glomeruli [88]. This study also showed that the topographical arrangement of aesthetascs along the distal–proximal extension of the first antenna did not map somatotopically to the olfactory glomeruli [88], indicating an odotopic rather than somatotopic representation of sensory input in the OL [14]. Our findings in *P. hawaiensis* support the odotopic representation hypothesis because both proximal and distal backfills labelled most olfactory glomeruli, though fewer fibres were stained in the distal backfill.

In decapod crustaceans, the antenna 1 neuropil (LAN) was shown to be primarily innervated by bimodal and mechanosensory sensilla [19,32,86,89]. The bifurcated structure of the first antenna likely aligns with the bilobed shape of the LAN, which was suggested to display a somatotopic organization so that the spatial arrangement of sensilla in the periphery is topically mapped onto this neuropil retaining the spatial relationship of neighbouring structures [32,89]. Axonal projections from the lateral and medial flagella of the first antennae in decapod crustaceans typically innervate the lateral and medial LAN, respectively [86]. In *P. hawaiensis*, in addition to the bilobed structure, we observed a striated pattern in both proximal and distal mass backfills of the LAN. In *P. argus* and *Procambarus clarkii*, backfills of the first antennal nerve revealed spatially distinct regions within the LAN, often with striated patterns, reinforcing the idea of somatotopic representation [86,89]. Additionally, the length of the LAN has been proposed to correlate with the length of the represented appendage [90,91]. However, our results do not fully support this hypothesis for *P. hawaiensis*. Although a striated pattern was observed in the LAN, both proximal and distal backfills filled the entire LAN rather than generating the expected partial staining in the distal backfill. This suggests that mechanosensory and chemosensory neurons from the tip of the first antennae may project throughout the full length of the LAN, potentially reflecting the importance of sensory input from the first antennae tip. While a somatotopic representation may still exist, our mass backfill approach did not reveal it in *P. hawaiensis*.

### 4.4. The numbers of olfactory glomeruli in *Parhyale hawaiensis* are characterized by a high level of inter-individual variation

Basal Malacostraca typically possess fewer than one hundred olfactory glomeruli: approximately 50 in *P. hawaiensis* [47,52] (also this study), approximately 35 in Mysidacea [92,93] (K.K. *et al*. 2024, unpublished data), approximately 60 in Leptostraca [94], approximately 80 in marine Isopoda [72], between 60 and 80 in Stomatopoda [95], and approximately 90 in Stenopodidea [96]. However, there is an evolutionary trend towards much higher glomerular numbers (often exceeding 1 000) in decapod crustaceans [10,75]. One remarkable result of our study is the finding that glomerular numbers are highly variable across the population of samples that we counted. What is more, we had expected the relative simplicity of the system and low numbers of olfactory glomeruli to facilitate recognizing individual identities of glomeruli. However, across the numerous olfactory glomeruli that we reconstructed in *P. hawaiensis*, none could be identified individually by size, their spatial position or neighbouring structures. In fact, so far, we do not know of any individually identifiable glomeruli in none of the malacostracan species studied so far (reviewed in [10]).

In the well-described decapod crustaceans, the glomeruli typically are elongated and cone shaped [10,14,25–30]. In contrast, spherical glomeruli dominate in all other malacostracan crustaceans examined to date [52,72,92,93]. Because Remipedia, as an out-group to Malacostraca also exhibit spherical glomeruli [97], it was suggested that spherical glomeruli are part of the ground pattern in these taxa, similar to hexapods [52].

### 4.5. The olfactory system of hexapods displays both similarities and differences to that in Malacostraca

**Figure 10.**
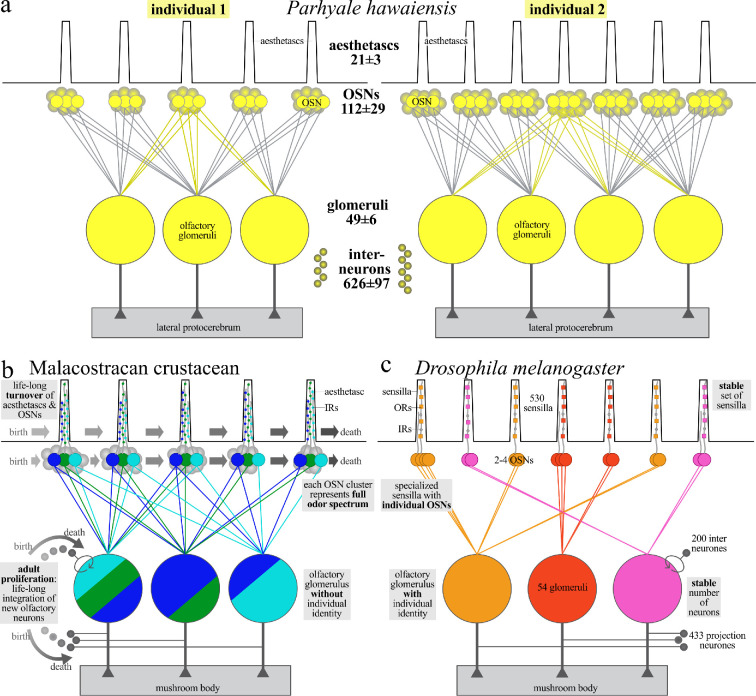
Olfactory wiring logic in crustaceans versus flies. (a) Schematic representation of the variability in the olfactory pathway between two *Parhyale hawaiensis* individuals as encountered in this study. The aesthetascs, the OSNs, the connections of the OSNs to the olfactory glomeruli, the connection of the glomeruli to the lateral protocerebrum and the interneurons which form the olfactory glomeruli are shown. Note that the link to the protocerebrum was not analysed in this study, but the information is derived from source [47]. (b) Schematic representation of the olfactory wiring in a malacostracan crustacean as derived from data in the present study and the following literature resources: general anatomy [10–12,14,25–28], olfactory receptor molecules [44,45], lifelong turnover of sensilla [79,80,98], adult generation of interneurons [99–102]. (c) Schematic representation of the one-to-one olfactory wiring principle in the hexapod *Drosophila melanogaster*. The sensilla can be subdivided into three major classes sensilla trichoidea, sensilla basiconica and sensilla coeloconica, and each sensillum is associated with the dendrites of between one of the four OSNs [103,104]. The colour code shows the connection from one type of receptor molecule per olfactory sensory neuron to one glomerulus with a defined identity. Derived from the following literature resources: general anatomy and wiring logic [36–42,103,105–108], sensilla [103,104,109–112], olfactory receptor molecules [113,114]. For further details, see section 4.5 Abbreviations: IR, ionotropic receptor molecule; OR, odorant receptor molecule; OSN, olfactory sensory neurons.

Going back to a common ancestor, hexapods and malacostracans exhibit several neuroanatomical similarities in their olfactory pathways, such as the general information flow from OSNs to olfactory glomeruli to projection neurons that target the mushroom bodies (summarized in figure 10 and [10]). The olfactory pathways of *P. hawaiensis* and of the vinegar fly *D. melanogaster* feature roughly similar numbers of neuronal elements: approximately 1 200 OSNs that project onto approximately 50 olfactory glomeruli that are innervated by approximately 600 olfactory interneurons. However, notable differences do also exist between hexapods and malacostracans related both to the peripheral and central olfactory pathway (reviewed in [10,35]; summarized in figure 10). For example, the olfactory receptor molecules embedded in the cell membrane of OSNs in marine crustaceans are predominantly IRs, derived from ionotropic glutamate receptors and consist of two classes: co-receptor IRs (e.g. IR25a and IR93a) and tuning IRs [23,113]. Hexapods have expanded their receptor repertoire as an apomorphic adaptation to terrestrial and aerial environments by the evolution of distinct seven-transmembrane odorant receptors (ORs) that likely emerged after this lineage conquered land and facilitated rapid odour detection in flight. Furthermore, hexapods evolved olfactory binding proteins and the olfactory co-receptor Orco, enhancing odour detection in air [113–115]. Pronounced differences also exist concerning the inventory of chemosensory sensilla on the pair of deutocerebral antennae of hexapods versus malacostracan crustaceans, as discussed in a comparative approach by Hallberg & Hansson [21]. The chemosensory sensilla of hexapods exhibit a large diversity of shapes and structures and include the three major classes sensilla trichoidea, sensilla basiconica and sensilla coeloconica [reviews 109–112,116], and currently, there is not any indication which of these three types may correspond to crustacean aesthetascs [18,21] (A. Steinbrecht 2018, personal communication). What is more, evidence obtained in decapod crustaceans indicates pronounced adult neuroplasticity in their peripheral olfactory pathways. In spiny lobsters, for example, the first antennal tuft has three zones responsible for generating, maintaining and shedding sensilla, corresponding with lifelong antennal growth and OSN turnover (figure 10b) [79,80,98]. In conclusion, several aspects of the peripheral olfactory pathway of hexapods are highly modified when compared with malacostracans, suggesting different coding strategies between these two taxa already in the periphery.

In the well-studied vinegar fly *D. melanogaster* the OSNs expressing the same odorant receptor molecules converge onto one olfactory glomerulus. Individual olfactory glomeruli are readily identifiable across individuals based on their spatial and morphological characteristics [36–38,103,105–107]. Similarly, individually identifiably olfactory glomeruli are observed in hexapods such as, e.g. the moth *Heliothis virescens* [117] and the red flour beetle *Tribolium castaneum* [118]. Furthermore, it was suggested that the number of olfactory glomeruli directly mirrors the diversity of OR input to the system, and because in many hexapods the number of identified OR genes roughly matches the number of glomeruli, a one-to-one wiring logic from receptor molecule to glomerulus was suggested to be the general rule for hexapods (figure 10), a system that enables combinatorial coding, wherein the activation of specific subsets of glomeruli corresponds to the perception of distinct odours (reviews in [39–42,108,119]). However, recent findings have suggested that in *D. melanogaster*, certain glomeruli show mixed odour representation from specific olfactory receptors [120,121] and that, in general, there is a larger diversity of olfactory coding in hexapods. For example, in *Aedes aegypti*, multiple glomeruli receive inputs from olfactory sensory neurons expressing different receptor types, creating mixed odour representation [122]. Another prominent example of an evolutionary transformation of antennal lobe morphology towards a non-canonical organizational logic comes from the Orthoptera (crickets and locusts; review in [123]). The desert locust *Schistocerca gregaria*, for instance, has densely packed microglomeruli targeted by multiple OSNs and projection neurons (PNs). In *Locusta migratoria*, physiological data indicate ring-shaped odour coding patterns [124].

Pronounced adult neuroplasticity does not only characterize the peripheral olfactory pathways of decapod crustaceans, but lifelong neurogenesis has also been documented in the central olfactory pathway of several malacostracan species, with the consequence of ongoing generation and integration of both local olfactory interneurons and projection neurons (figure 10b) [14,99–102]. In these animals, the number of olfactory glomeruli is variable among individuals, and so far, we have no evidence for individually identifiable glomeruli ([10], present study). It would appear that Malacostraca lack several neuroanatomical features that may be considered essential for a one-to-one olfactory wiring logic from receptor to glomerulus such as a highly stable numbers of neuronal elements and individually identifiable structures across individuals. Therefore, it was suggested that odour coding in malacostracans relies on a distributed representation of olfactory stimuli across glomerular populations which lack individual identities [10]. If we assume this to represent the ancestral state for Pancrustacea, which was transformed during evolution into the derived olfactory wiring logic that we see today in the fly and other insects (figure 10), we must ask which distinct steps this transformation may have required. These may have included the following:

(1) stop lifelong turnover and establish a stable set of sensilla with OSNs that express specific ORs.
(2) stop lifelong neurogenesis in the central olfactory pathway to establish a stable population of olfactory interneurons.
(3) tune the developmental programme such as to generate a fixed number of olfactory glomeruli with individual identities.
(4) find molecular mechanisms to guide all afferents that express one receptor type to one glomerulus.

## Data Availability

The data that support the findings of this study are available from the corresponding author upon reasonable request. Supplementary material is available online [125].
